# A New Heavy-Duty Bearing Degradation Evaluation Method with Multi-Domain Features

**DOI:** 10.3390/s24237769

**Published:** 2024-12-04

**Authors:** Ruolan Xiong, Aihua Liu, Dongfang Xu, Chunyang Qu, Yulong Wu

**Affiliations:** 1 School of Naval Architecture, Ocean and Energy Power Engineering, Wuhan University of Technology, Wuhan 430070, China; 2 Sanya Science and Education Innovation Park, Wuhan University of Technology, Sanya 572024, China

**Keywords:** heavy-duty bearing, multi-domain feature, initial degradation time, quantitative assessment of degradation

## Abstract

Under heavy load conditions, bearings are subjected to non-uniform and frequently changing loads, which leads to randomness in the spatial distribution of bearing degradation characteristics. Aiming at the problem that the traditional degradation index cannot accurately reflect the degradation state of heavy-duty bearings in the whole life cycle, a new degradation evaluation method based on multi-domain features is proposed in this paper, which aims to capture the early degradation point of heavy-duty bearings and characterize their degradation trend. Firstly, the energy entropy feature is obtained by improving the wavelet packet decomposition, and the original multi-domain feature set is constructed by combining the time domain and frequency domain features. Then, the optimal feature matrix is formed by using the comprehensive evaluation index. Finally, integrating probability and distance information, a comprehensive degradation index was constructed to evaluate the degradation, determine the initial degradation time, and quantitatively analyze the bearing degradation state. The validity of the proposed method is verified in two datasets. The proposed method can accurately identify the early degradation of bearings and track the state of bearing degradation, so as to realize the degradation assessment.

## 1. Introduction

Rolling bearings are the most common failure parts in mechanical equipment [1], and their failures account for about 45%-55% of the total failures of mechanical equipment [2]. Consequently, the degradation assessment of bearings is very important for the operation of equipment. The core of the degradation assessment is the establishment of the degradation index (DI) [3,4], which is used to accurately quantify the state of equipment degradation, identify early degradation points (EDPs) and analyze degradation trends. This enables timely preventive measures to be taken before a significant decline in equipment performance occurs, helping to prevent accidents, achieve predictive maintenance, and improve the safety and maintainability of the equipment [5,6]. However, under heavy load conditions, bearings are subjected to non-uniform and frequently changing loads, which makes the spatial distribution of degradation characteristics random, and it is difficult to accurately evaluate the bearing degradation state. How to effectively assess the degradation of heavy-duty bearings has become one of the research hotspots in the field of equipment health management.

DI construction based on statistical features is one of the common degradation evaluation methods [7], and its typical representatives include root mean square (RMS) and kurtosis. However, studies [8] show that the signal disturbance in the early degradation stage is relatively slight, and traditional statistical methods have insufficient ability to capture weak features, resulting in a low success rate of degraded state detection. Therefore, in order to improve the sensitivity of early degradation, scholars conducted further studies. For example, Yuan et al. [9] combined kurtosis and pulse index to obtain the overall DI; Rai et al. [10] combined multi-scale fuzzy entropy and Jensen–Renyi scatter to construct a new health index; and Hu et al. [11] used wavelet transform and other methods to achieve bearing degradation assessment. However, the degradation information contained in a single domain feature is small and incomplete, which affects the accuracy of EDP identification and degradation state analysis. In order to solve the problem of insufficient information, it has become an important method to extract multi-domain features and introduce feature evaluation criteria. For example, Wu et al. [12] used Pearson correlation coefficient to screen 10 groups of time domain statistical features, and Liu et al. [13] used Spearman coefficient to select the features obtained after Hilbert–Huang transformation. Tang et al. [14] fused multi-domain features and combined the weighted sum of correlation, monotonicity and robustness to select features; Yang et al. [15] fused time domain, frequency domain and wavelet decomposition energy features and used the average value of trend and monotonicity to screen sensitive degradation features from multi-domain features. The high-dimensional degradation feature set is formed by selecting features which can reflect the degree of bearing degradation.

With the increasing sensitivity and accuracy of degradation identification, the traditional methods based on statistical features have some problems such as inaccurate characterization of degradation trend and poor generalization ability when facing the challenge of random distribution of heavy-duty bearing characteristics. In order to overcome these limitations, scholars began to pay attention to the probability density method and boundary distance method. These methods can improve the accuracy and sensitivity of degradation analysis, so as to realize a more comprehensive understanding and quantification of degradation phenomena. The method based on probability density can reduce the influence of random distribution of degradation features and improve the accuracy of analysis. Among them, the Gaussian mixture model (GMM) [16,17], as a typical probability density method, uses probability distribution to infer data characteristics and relationships. For example, Hong et al. [18] used GMM to evaluate rolling bearings and realized early fault detection of bearings. Yu et al. [19] used the negative log-likelihood probability (NLLP) of GMM output to evaluate bearing degradation and identify minor bearing degradation. However, these methods have the problem of premature saturation, resulting in a certain gap between the degradation curve and reality, so there are still limitations. On the other hand, the degradation curve obtained by the method based on boundary distance is close to reality, which quantifies the degree of degradation by calculating the distance between the data and the set boundary. For example, Wu et al. [20] proposed constructing a principal component Mahalanobis distance (MD) health index based on characteristic data. Wei et al. [21] used Jensen–Shannon (JS) divergence to detect distribution differences and realize degradation assessment. Sun et al. [22] measured the degree of degradation by combining improved kernel Mahalanobis distance and fuzzy C-mean (FCM). Zhou et al. [23] proposed a degradation index based on wavelet packet decomposition (WPD) and fuzzy C-mean (FCM), using the wavelet packet node energy as the feature vector, for degradation assessment. While these methods are able to track bearing degradation, especially at the end of the bearing’s life, they are not effective at identifying early degradation.

Based on the above research, this paper proposes a new degradation assessment method based on multi-domain features. Through multi-domain feature extraction and fusion degradation index construction, EDP recognition and degradation trend characterization are realized. This thesis mainly involves the following contents:

(1) Improved wavelet packet for multi-domain feature extraction. This paper innovatively introduces JS divergence to optimize the level of wavelet packet decomposition to reduce information loss and obtain a better decomposition effect. At the same time, combining the time domain and frequency domain features, the original high-dimensional feature set was constructed, and the features were screened based on the comprehensive evaluation index to reduce the interference of redundant features and fully reflect the running state of the bearing.
(2) A new degradation assessment method was proposed. In this method, probability density information was introduced by GMM, and the boundary distance information of FCM and MD was combined to generate DI with multi-dimensional information, which was used to evaluate the degradation state of bearings. DI was used to measure the difference between the observed degradation state and the known degradation mode, identify EDP, and comprehensively reflect the degradation process.
(3) The experiment verified the superiority of the proposed method. In this paper, the degradation data of 18 bearings in two datasets were used to verify the method, and the effectiveness and accuracy of the method were verified by comparing with the EDP recognition results in other studies. In addition, the monotonicity and trend were considered in the construction of the degradation assessment index, and comparison with other methods further proved the effectiveness and comprehensiveness of the proposed method.

The remaining structure of this paper is as follows: Section 2 introduces the theoretical methods of this paper. Section 3 describes the proposed method in detail. Section 4 verifies the effectiveness of the proposed method through two datasets, and Section 5 summarizes the whole paper.

## 2. Related Works

### 2.1. Multi-Domain Feature Extraction

Commonly used feature extraction methods include the following: time domain feature extraction method, frequency domain feature extraction method, time–frequency domain feature extraction method. In the time domain, different statistical features are extracted to represent how the signal amplitude changes with time. In the frequency domain, features in a given frequency band are extracted to detect the corresponding fault characteristics of the frequency component [24].

Sixteen commonly used time domain features and four commonly used frequency domain features were selected to reflect the fault state of rolling bearings, and the calculation formula [25] is shown in Table 1 and Table 2, where x(n) is n=1,2,…,N is the signal sequence, where *N* is the number of sample points. For the time series signals $x_{1},x_{2},\ldots ,x_{N}$ and the frequency domain amplitude spectrum reference, Equation (1) is obtained by Fourier transform:

(1) $$\;\;s\left(k\right)=\sum_{i=1}^{N}x_{i}e^{-j2\pi (i-1)(k-1)/N}$$

where s(k) is the spectrum for k=1,2,…,N, *N* is the number of spectral lines, and $f_{k}$ is the frequency value of the *k* spectral line.

Because the original vibration signal of rolling bearing is a non-stationary signal, it is difficult to accurately characterize the change rule of the original signal by using only the time domain and frequency domain characteristics, so it is necessary to introduce time–frequency analysis to analyze the original vibration signal of rolling bearing. This paper uses wavelet packet decomposition (WPD), a time–frequency domain analysis technique, to extract energy characteristics and describes the change in energy distribution based on energy entropy [26], namely, wavelet packet energy entropy (WPEE) feature. Wavelet packet energy entropy [27] decomposes the vibration signal through a wavelet packet to obtain the decomposition coefficient and reconstruction coefficient, so as to calculate the characteristic energy of the reconstruction coefficient, then calculate the ratio of the energy of each node to the total energy, and finally obtain the information entropy of the characteristic energy ratio, which combines the concepts of wavelet transform and information entropy. It is used to analyze and understand the characteristic information in complex vibration signals for more efficient degradation analysis.

Suppose $E_{1},E_{2},\ldots ,E_{k}$ is the energy spectrum of the signal at the decomposition scale *k*, and the sum of energy $E_{i}\left(i=1,2,\ldots ,k\right)$ of the points is equal to the total energy *E* of the signal, and the proportion of energy at each scale is $\varepsilon_{jk}\left(i\right)=E_{i}/E$, and $\sum_{k}\varepsilon_{jk}\left(i\right)=1$. According to the definition of WPEE, the calculation formula for the energy entropy feature of the wavelet packet is as follows [28]:

(2) $$\;\;S_{jk}=-\sum_{i=1}^{N}\varepsilon_{jk}\left(i\right)lg\varepsilon_{jk}\left(i\right)$$

where *N* is the original signal length, and $S_{jk}$ represents the wavelet packet energy entropy of the $k_{th}$ component of the signal at the $i_{th}$ level.

When performing wavelet packet decomposition, the key is to determine the appropriate decomposition scale in order to effectively distinguish between normal vibration data and failure vibration data, while avoiding overfitting and noise interference. Jensen–Shannon (JS) divergence focuses on the overall difference between two distributions, measures the similarity or difference between two probability distributions, and can better capture the difference between features.

Given two probability distributions *P* and *Q* and the average distribution of the two M=1/2(P+Q), their JS divergence calculation formula [29] is defined as follows:

(3) $$JS\left(P||Q\right)=\frac{1}{2}\sum_{i}\left[P\left(i\right)log\left(\frac{P(i)}{M(i)}\right)+Q\left(i\right)log\left(\frac{Q(i)}{M(i)}\right)\right]$$

**Figure 1:**
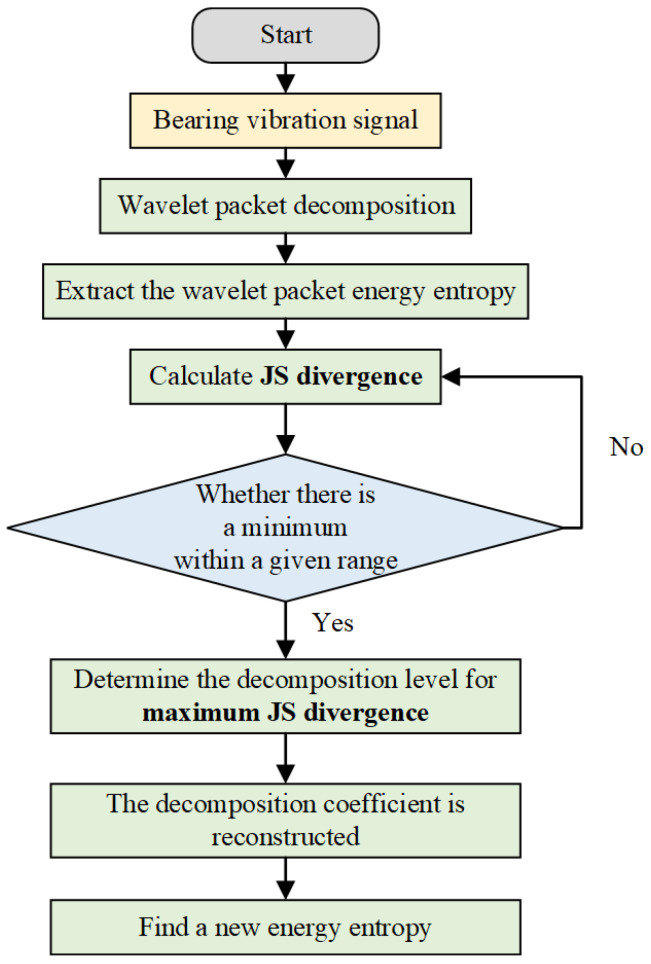
Steps of improved wavelet packet energy entropy feature extraction.

In order to improve the accuracy of degradation evaluation, JS divergence is introduced to improve the wavelet packet decomposition method. The specific process is shown in Figure 1. Firstly, the original vibration signal is decomposed by a wavelet packet in the range of the proposed decomposition level, and the JS divergence between the normal vibration data and the failure vibration data is calculated under each decomposition level. Then, the decomposition level with the largest difference is selected as the best level. Finally, the energy entropy feature is extracted under this optimal level. Doing so can more accurately distinguish between normal and invalid data, significantly enhancing the effectiveness of anomaly detection.

### 2.2. Feature Optimization

To perform feature selection, it is first necessary to establish evaluation criteria for relevant degradation features. These criteria are used to assess the results of feature extraction, discarding redundant features that are unrelated to the degradation process while retaining features that can represent degradation trends, thus facilitating more effective degradation assessment. Based on practical engineering analysis and related studies [30,31], three indicators for feature selection were determined: monotonicity (Mon), correlation (Corr), and robustness (Rob). A comprehensive evaluation metric was then established using these three indicators to measure the degree of association between the bearing degradation features and the time series. This allows for a more accurate analysis of the changes in bearing health conditions and provides a certain level of resistance to outliers and noise interference, offering better stability. The specific steps are as follows [20]:

(1) To address the uncertainty of degradation features, a smoothing analysis method was used to process the degradation features. The exponential weighted moving average (EWMA) method was applied to divide the degradation features into two parts (trend and randomness):
  (4) $$\;\;f\left(t_{k}\right)=f_{T}\left(t_{k}\right)+f_{R}\left(t_{k}\right)$$
  (5) $$\;f_{T}\left(t_{k}\right)=\beta f_{T}\left(t_{k-1}\right)+\left(1-\beta \right)f_{T}\left(t_{k}\right)$$
  where f(t) is the degenerate eigenvalue; $f_{T}\left(t\right)$ is the trend value part; and $f_{R}\left(t\right)$ is the random feature part.
(2) The features’ Mon, Corr, and Rob were calculated as follows:
  (6) $$\left\{\begin{array}{cc} Corr= & \frac{|\sum_{k=1}^{K}\left(t_{k}-\bar{t}\right)\left(f\left(t_{k}\right)-\bar{f(t)}\right)|}{\sqrt{\left[\sum_{k=1}^{K}{\left(t_{k}-\bar{t}\right)}^{2}\right]\left[\sum_{k=1}^{K}{\left(f\left(t_{k}\right)-\bar{f(t)}\right)}^{2}\right])}}\\ Mon= & \frac{\sum_{k=1}^{K}\delta \left(f_{T}\left(t_{k+1}\right)-f_{T}\left(t_{k}\right)\right)-\sum_{k=1}^{K}\delta \left(f_{T}\left(t_{k}\right)-f_{T}\left(t_{k+1}\right)\right)}{K-1}\\ Rob= & \frac{1}{K}\sum_{k=1}^{K}\exp\left(-|\frac{f_{R}\left(t_{k}\right)}{f(t_{k})}|\right) \end{array}\right.$$
  where δ(t) is a unit step function, specifically expressed as follows: $\delta \left(t\right)=\left\{\begin{array}{cc} 1, & t\geq 0;\\ 0, & t\leq 0. \end{array}\right.$
  The value range for the above three feature evaluation indicators is [0, 1]. The higher the value, the stronger the correlation, monotonicity, or robustness. Before calculating the three feature evaluation indicators, the input data need to be normalized.
(3) A weighted linear combination was constructed to integrate the three evaluation indicators, which served as the final independent evaluation criterion. It is specifically expressed as follows:
  (7) $$Cri=\omega_{1}Corr+\omega_{2}Mon+\omega_{3}Rob$$
  (8) $$s=\left\{\begin{array}{cc} \omega_{i}> & 0\\ \sum_{i}\omega_{i}= & 1 \end{array}\right.$$
  where Cri of Equation (7) is the comprehensive objective optimization function and, $\omega_{i}\left(i=1,2,3\right)$ is the evaluation index coefficient. Equation (8) represents the constraint conditions of weight coefficient $\omega_{i}$, and $\omega_{i}>0$ ensures the positive contribution of all indicators to the comprehensive evaluation and $\sum_{i}\omega_{i}=1$ in order to unify the scale of Cri, avoid weight imbalance, and facilitate the comparison of indicator contributions. The three evaluation indexes were combined to obtain the comprehensive index Cri, and the sensitive degradation features were screened by Cri. The higher the Cri score of the degradation features, the more the degradation features could reflect the degradation information of bearings. This paper sets the coefficient vector $\left(\omega_{1},\omega_{2},\omega_{3}\right)=\left(0.3,0.4,0.3\right)$ according to the knowledge and experience of domain experts.

### 2.3. Gaussian Mixture Model (GMM)

The probability-based method is an important method for solving the stochastic problem of feature distribution by obtaining the degree of similarity between healthy samples and whole-life samples using probabilistic models. Among them, GMM [16,17] is the most widely used. First, we assume that the data $x={\left(x_{1},x_{2},\ldots ,x_{N}\right)}^{T}$ obey the Gaussian mixed distribution, and $x_{i}$ is an m-dimensional random variable. Then, the probability model is as follows:

(9) $$\;\;\;H\left(x_{i}|\theta \right)=\sum_{k=1}^{K}p_{k}h\left(x_{i}|\mu_{k},\Sigma_{k}\right)$$

where $h(x_{i}|\mu_{k},\Sigma_{k})$ is the component probability density function; θ represents the parameter set of each submodel in GMM and is a function of μ and Σ; $p_{k}$ represents the probability that the sample comes from this component model, that is, it represents the probability of the $k_{th}$ submodel appearing in the population $\sum p_{k}=1$; *K* is the number of Gaussian distributed components (the number of Gaussian distributions), representing a total of *K* submodels. The probability density function of a multivariate Gaussian distribution is defined by the Equation (10):

(10) $$h\left(x_{i}|\mu_{k},\Sigma_{k}\right)=\frac{1}{{\left(2\pi \right)}^{\frac{m}{2}}{\left|\Sigma_{k}\right|}^{\frac{1}{2}}}\exp\left(-\frac{1}{2}{\left(x_{i}-\mu_{k}\right)}^{T}\Sigma_{i}^{-1}\left(x_{i}-\mu_{k}\right)\right)$$

where $x_{i}$ is the eigenvector; $\mu_{k}$ is the mean vector of the $k_{th}$ component; *m* is the dimension of the feature $x_{i}$; $\Sigma_{k}$ is the covariance matrix of the $k_{th}$ component; $|\Sigma_{k}|$ is the determinant of the covariance matrix of the $k_{th}$ component; and $\Sigma_{k}^{-1}$ is the inverse of the covariance matrix of the $k_{th}$ component.

Maximum likelihood estimation is used to find the mean and variance of each submodel. For *N* independent equally distributed samples and the given hyperparameter *K*, the likelihood function of the distribution parameter is as follows:

(11) $$L\left(\theta \right)=ln\prod_{i=1}^{N}H\left(x_{i}|\theta \right)=\sum_{i=1}^{N}\left(ln\sum_{k=1}^{K}p_{k}h\left(x_{i}|\mu_{k},\Sigma_{k}\right)\right)$$

For the parameters in GMM, the Expectation Maximum (EM) algorithm can be used to estimate the parameters in GMM iteratively. The EM algorithm steps are as follows:

(1) Let the training set be $x={\left(x_{1},x_{2},\ldots ,x_{N}\right)}^{T}$, $\theta =\{p_{k},\mu_{k},\Sigma_{k}\}$ for GMM parameter θ estimates using the maximum likelihood estimation method, i.e., solving the maximum likelihood function L(θ). The parameter estimation problem is transformed into
  (12) $$\;\;\;\hat{\theta }=\arg\max\left(L(\theta )\right).$$
(2) According to Bayes theory, the posterior probability $\beta_{k}^{\left(t\right)}\left(x_{i}\right)$ can be expressed as
  (13) $$\;\beta_{k}^{\left(t\right)}\left(x_{i}\right)=\frac{p_{k}^{\left(t\right)}h\left(x_{i}|\mu_{k},\Sigma_{k}\right)}{\sum_{k=1}^{K}p_{k}^{\left(t\right)}h\left(x_{i}|\mu_{k},\Sigma_{k}\right)}$$
  where *t* represents the $t_{th}$ iteration.
(3) The maximization process is as follows:
  (14) $$\left\{\begin{array}{cc} p_{k}^{\left(t+1\right)}= & \frac{1}{N}\sum_{i=1}^{N}\beta_{i}^{\left(t\right)}\left(x_{i}\right)\\ \mu_{k}^{\left(t+1\right)}= & \frac{\sum_{i=1}^{N}\beta_{k}^{\left(t\right)}\left(x_{i}\right)x_{i}}{\sum_{i=1}^{N}\beta_{k}^{\left(t\right)}\left(x_{i}\right)}\\ \Sigma_{k}^{\left(t+1\right)}= & \frac{\sum_{i=1}^{N}\beta_{k}^{\left(t\right)}\left(x_{i}\right){\left(x-\mu_{k}^{\left(t+1\right)}\right)}^{T}\left(x_{i}-\mu_{k}^{\left(t+1\right)}\right)}{\sum_{i=1}^{N}\beta_{k}^{\left(t\right)}\left(x_{i}\right)} \end{array}\right.$$
  where the $p_{k}^{\left(t+1\right)}$, $\mu_{k}^{\left(t+1\right)}$, $\Sigma_{k}^{\left(t+1\right)}$ after (t+1) iterations are, respectively, the weight of the first *k* Gaussian distribution, the mean, and covariance matrix. By repeating these three formulas multiple times, iterating until the likelihood function converges, the GMM model parameters are obtained.

In particular, the mixed number (n components) needs to be defined in advance, so this paper adopted the Bayesian Information Criterion (BIC) [32], and the optimal number of mixed models is the value when the BIC score is lowest. BIC pays more attention to punishing model complexity, and points with low BIC scores usually have good model complexity and data fit. BIC scores are defined as follows:

(15) BIC=Kln(n)−2ln(L)

where *K* is the number of estimated parameters; *n* is the number of observations; *L* is the likelihood function. After the GMM model was established, negative log-likelihood probability (NLLP) was used to measure the degree of normal (or abnormal), which reflects the similarity between the data to be evaluated and the normal state, defined as follows:

(16) $$\;\;NLLP=-\log\left(x|\hat{\theta }\right)$$

where *x* represents the data point to be evaluated, and $\hat{\theta }$ represents the estimated parameters of the GMM (including mean, covariance, and mixing coefficient).

### 2.4. Fuzzy C-Means Algorithm Based on Mahalanobis Distance

#### 2.4.1. Fuzzy C-Means (FCM)

Fuzzy C-mean is a clustering algorithm that determines the sample belongs to a certain class by membership degree. Membership degree ranges from [0,1], which can represent the degree of deviation from normal state of the sample to be tested. Setting the data sample of $x=\left(x_{1},x_{2},\ldots ,x_{n}\right),c\left(2≦c≦n\right)$ to the sample into the category of the number, $A=(A_{1},A_{2},\ldots ,A_{c})$ gives the corresponding *c* category, and all kinds of other clustering centers are $C=(\nu_{1},\nu_{2},\ldots ,\nu_{c})$, where $u_{ik}$ is the membership of the sample $x_{i}$ for class $A_{k}$, and $U=[u_{ik}]$ is the fuzzy membership matrix. In the FCM algorithm, the membership degree sum of a certain sample $x_{i}$ for each category is 1, that is,

(17) $$\;\;\sum_{k=1}^{c}u_{ik},i=1,2,\ldots ,n.$$

The implementation process of the FCM clustering algorithm is as follows [22,33]:

(1) Parameter initialization. Set the initial values of the number of categories *c* and the fuzzy parameter *q*, and initialize the cluster center matrix *C*. Update the cluster center, cluster center $\left\{\nu_{k}\right\}$ published as follows:
  (18) $$\;\;\nu_{k}=\frac{\sum_{i=1}^{n}{\left(u_{ik}\right)}^{q}x_{i}}{\sum_{i=1}^{n}{\left(u_{ik}\right)}^{q}}$$
(2) Update the fuzzy membership matrix U. Calculate the membership $u_{ik}$ of sample $x_{i}$ for class $A_{k}$:
  (19) $$\;\;u_{ik}=\frac{1}{\sum_{j=1}^{c}{\left(\frac{d_{ik}}{d_{ij}}\right)}^{\frac{2}{q-1}}}$$
  where $d_{ik}=\left|\right|x_{i}-\nu_{k}\left|\right|$, $d_{ij}=\left|\right|x_{i}-\nu_{j}\left|\right|$, i.e., the Euclidean distance is used to measure samples with absolute distance clustering center.
(3) If the difference between two adjacent clustering values is less than the set initial threshold, stop the iteration; otherwise, return to (2).

#### 2.4.2. MD-Based FCM Algorithm

Since Mahalanobis distance (MD) considers the correlation between features, it can better measure the similarity between features and can better adapt to the distribution characteristics of data; this paper introduces MD into the traditional FCM algorithm, replaces the Euclidean distance function with the MD function [34], and obtains the FCM clustering model based on MD (FCMD model). The expected loss error of measurement learning, cluster number of FCM and fuzzy parameters are set, and the Markov matrix $M_{\mathrm{normal}}$ and $M_{\mathrm{failure}}$ and cluster center are obtained by training data. The distance $d_{\mathrm{normal}}$ and $d_{\mathrm{failure}}$ between the state feature parameter x to be evaluated and the clustering centers $\nu_{\mathrm{normal}}$ and $\nu_{\mathrm{failure}}$ is calculated. According to the membership calculation formula, the distance is converted to the membership $u_{\mathrm{normal}}$ relative to the normal state, the formula is as follows:

(20) $$\left\{\begin{array}{cc} d_{\mathrm{normal}}= & \sqrt{\left(x-\nu_{\mathrm{normal}}\right)\sum^{-1}{\left(x-\nu_{\mathrm{normal}}\right)}^{T}}\\ d_{\mathrm{failure}}= & \sqrt{\left(x-\nu_{\mathrm{failure}}\right)\sum^{-1}{\left(x-\nu_{\mathrm{failure}}\right)}^{T}} \end{array}\right.$$

(21) $$\;\;u_{\mathrm{normal}}={\left(1+{\left(\frac{d_{\mathrm{normal}}}{d_{\mathrm{failure}}}\right)}^{\frac{2}{q-1}}\right)}^{-1}$$

where $u_{\mathrm{normal}}$ refers to the degree of normal state.

## 3. Proposed Method

**Figure 2:**
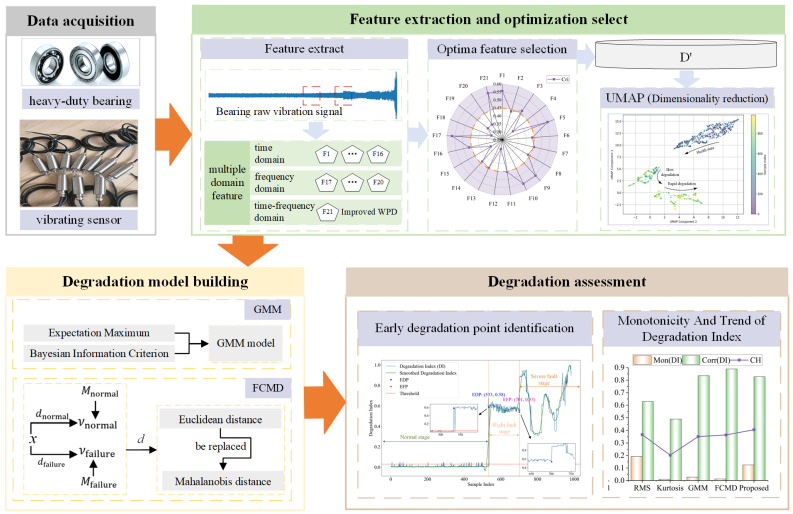
General frame diagram.

This paper proposes a new degradation assessment method based on fusion of multi-domain features. The overall framework is shown in Figure 2.

The specific description is as follows:

Step 1: Collect data. Collect bearing vibration signals of the engine, hydraulic system and other mechanical equipment under heavy load.
Step 2: Feature extraction and optimization. Calculate the time domain, frequency domain and time–frequency domain features of the original vibration signal for a single sample signal, and construct the original multi-domain feature set. Combined with monotonicity, correlation, and robustness, construct a comprehensive evaluation index, carry out feature screening, and select effective degradation features of a high-dimensional feature set to establish the preferred feature set.
Step 3: Build the degraded model. Use UMAP dimensionality reduction algorithm to reduce the dimensionality of each line vector of the feature matrix, fuse the extracted features, and use the two-dimensional matrix after dimensionality reduction to train the GMM model and FCMD model. Input the test set into the two trained models for fusion to obtain fusion DI.
Step 4: Degradation assessment. Identify EDP by setting a threshold value, evaluate DI performance, analyze the degradation curve to track the degradation stage, and achieve degradation assessment.

### 3.1. The Specific Process of the Proposed Method

**Figure 3:**
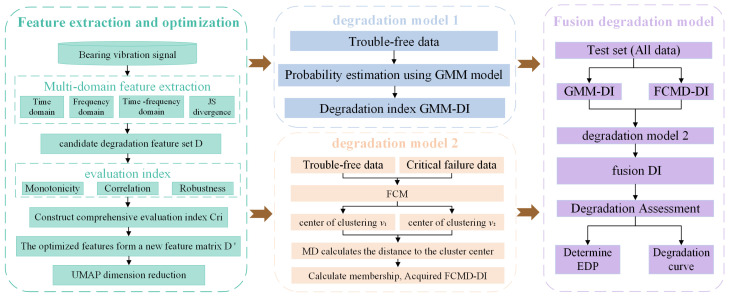
General frame diagram.

A new degradation assessment method is proposed in this paper. The specific process is shown in Figure 3:

(1) Feature extraction and optimization. The features are extracted from the original vibration data and normalized, the original multi-domain feature set is established, and the optimal feature set is constructed through optimization; then, dimensionality reduction is carried out.
(2) Training model. The GMM model is trained with normal fault-free data, and the model parameters are obtained. The FCMD model is trained to calculate the Markov matrix under the normal state and severe fault state, and the corresponding clustering center is obtained.
(3) Degradation assessment. The NLLP value of the test set is calculated by the GMM model, which is denoted as GMM-DI. By using the FCMD model, the MD between the test set and the training cluster class center is calculated, and the membership degree calculation formula is used to convert the MD to the membership degree relative to the normal state, and FCMD-DI is obtained. GMM-DI and FCMD-DI are re-input into the FCMD model as two columns of features, a new fusion index DI is obtained to identify EDP, and degradation curves are obtained to analyze the bearing degradation state and achieve degradation assessment.

### 3.2. Evaluation Index

For mechanical parts, EDP identification and degradation trend tracking are key technologies in the life cycle performance degradation evaluation of mechanical equipment [35], among which, the setting of threshold for early degradation detection and the selection of evaluation indicators for evaluation methods are particularly important.

#### 3.2.1. Threshold Setting

In order to monitor the degradation of components, two threshold strategies are set in this paper:

(1) The value of “threshold−1” can be set according to the 3σ principle, and its calculation formula is as follows [36]:
  (22) $$\;threshold-1=mean\left(1:t_{s}\right)\pm 3std\left(1:t_{s}\right)$$
  where $t_{s}$ is set to the number of samples corresponding to the running data when the bearing is healthy and trouble-free in early service. In the case of abandoning the abnormal discrete points and setting three consecutive deviations from the threshold range, the first deviation from “threshold−1” is taken as the beginning of the degradation stage, the EDP.
(2) DI gradient analysis [17]: The trend of DI is used to analyze the bearing degradation, as shown in Equation (23). When the gradient of DI (GDI) changes abruptly, it indicates that the bearing may be degraded or faulty.
  (23) $$\;\;GDI=\frac{\partial \mathrm{DI}}{\partial x}\hat{\iota }$$
  where *x* represents time, and $\frac{\partial \mathrm{DI}}{\partial x}\hat{\iota }$ represents the gradient of DI with respect to *x*. In order to identify the mutation point, this paper defines the threshold of “mutation point” as 0.05, and when GDI exceeds the threshold, it is considered that there is mutation at this point. To avoid misjudgment, if the threshold is exceeded three consecutive times, the first point is set as the start of the fault phase, marking it as the early failure point (EFP), and the horizontal line of this point is the “threshold−2” value.

#### 3.2.2. Evaluation Methods

In order to better assess degradation, this paper defined Composite Health (CH) to conduct a quantitative evaluation of DI performance, which combined monotonicity and trend. Among them, monotonicity was used to describe the monotonicity increase or decrease in the degradation process. Since degradation is irreversible, DI with a high Mon(DI) value can describe the degradation mechanism more clearly. Trend was reflected by measuring the correlation between DI and time. Here, Pearson correlation coefficient was used to measure the constructed linear correlation between DI and time, namely, Corr mentioned in Section 2.2 of this paper. The DI with high Corr(DI) value showed obvious trend and small fluctuation, which can more accurately characterize the degradation process. CH is defined as follows:

(24) $$\;CH=m_{1}Mon\left(DI\right)+m_{2}Corr\left(DI\right)$$

where Mon and Corr are defined in Equations (6).

## 4. Experiment

In this section, the superiority of the proposed method is verified by two experimental cases. In experiment 1, the ability of the proposed method to identify EDP and accurately distinguish different degradation stages is verified; At the same time, we pay attention to the monotonicity and tendency of DI, and compare it with the traditional and single method to further verify the effectiveness and comprehensiveness of the proposed method. In experiment 2, the ability of the proposed method in tracking degradation states is mainly investigated, and the two dimensions of monotonicity and trend are comprehensively evaluated. The specific analysis is as follows.

### 4.1. Experiment 1

**Figure 4:**
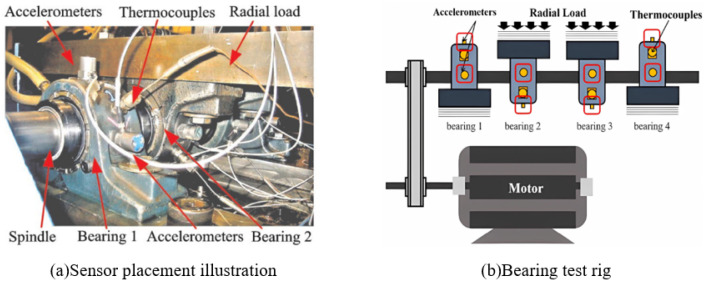
The rolling bearing test rig of IMS.

The experimental data came from the NSFI/UCR Intelligent Maintenance System Center (IMS) of the United States [37], and the experimental equipment is shown in Figure 4. The bearing speed was 2000 r/min, the experimental sampling frequency was 20.48 kHz, sampling interval was 10 min, and the theoretical fault frequency $f_{0}$ of the outer ring was 236 Hz. The specific experimental data are shown in Table 3.

**Figure 5:**
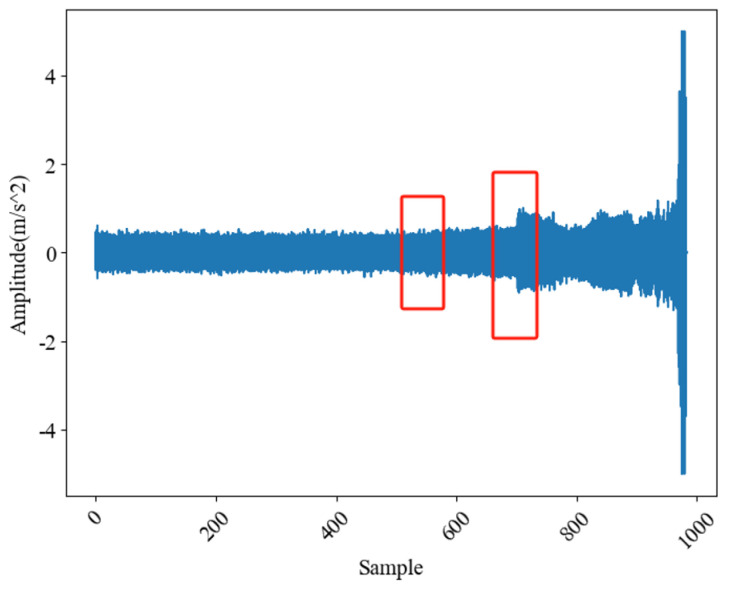
IB2−1 vibration time domain waveform.

Taking IB2-1 as an example, the time domain waveform of its whole life cycle data is shown in Figure 5. Only a general trend can be seen from the figure, that is, the first 500 groups of data are very stable and in a healthy state, while the amplitude fluctuation of the 500th to 600th group increases (the first red box in the figure), and the fluctuation is particularly obvious when the data of the 700th group are around (the second red box in the figure), and approximately, the last 10 groups are close to failure.

Since the fault characteristics of the vibration signal in the early degradation stage of the bearing are not obvious, and the running state of the bearing can not be clearly divided, the analysis needs to be combined with the signal processing method.

#### 4.1.1. Multi-Domain Feature Extraction and Dimensionality Reduction

**Figure 6:**
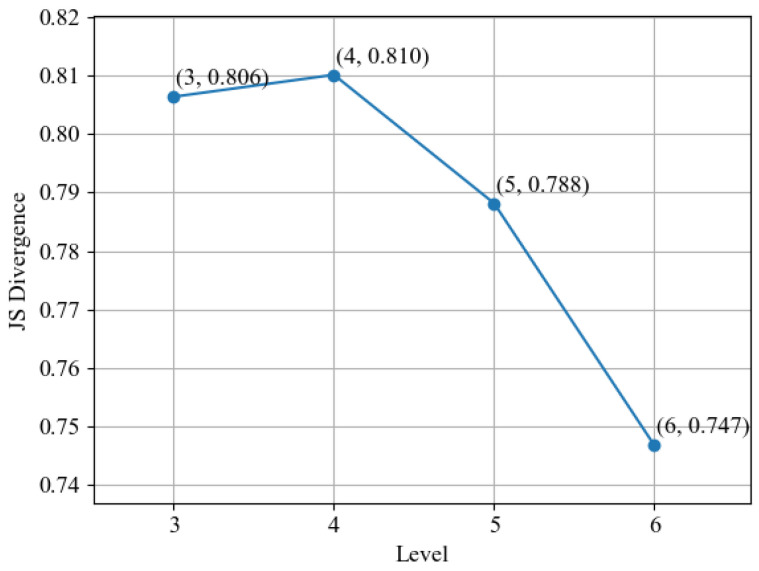
IB2-1 vibration time domain waveform.

**Figure 7:**
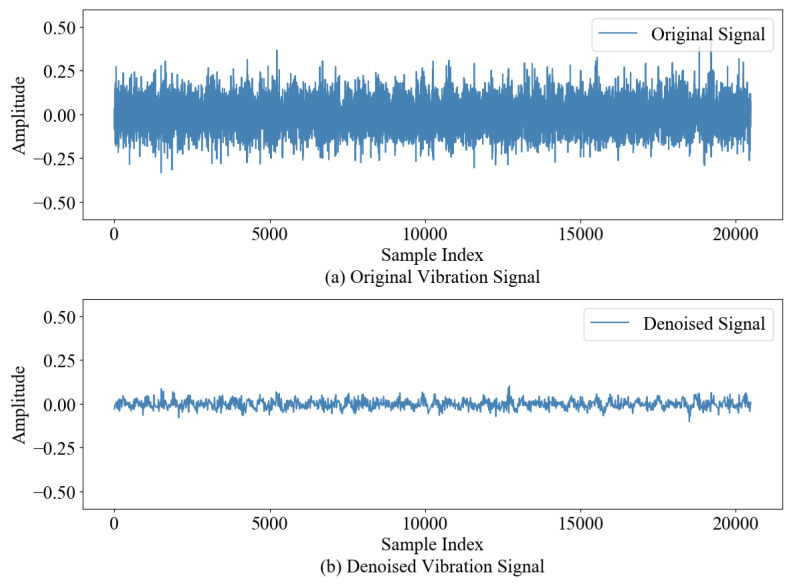
Comparison of vibration signals before and after wavelet packet decomposition.

In order to better distinguish the feature difference between normal data and failure vibration data and improve the accuracy and reliability of degradation analysis, this paper adopted JS divergence to measure the difference degree. The results are shown in Figure 6, where the horizontal axis is the decomposition level, and the vertical axis is the JS divergence value between the energy entropy of normal data and the energy entropy of failure vibration data. The decomposition levels were set to range from 3 to 6, and the JS divergence can be calculated to be about 0.806, 0.810, 0.788 and 0.747, respectively. It can be seen that the decomposition difference is the largest when level = 4. According to the theory in Section 2.1, the decomposition level is 4 to effectively distinguish between normal vibration data and failure vibration data. Figure 7 shows the comparison of vibration signals before and after wavelet packet decomposition using the optimal decomposition level. Among them, Figure 7a is the original signal of the 7th group of data, and Figure 7b is its denoised vibration signal. It can be seen that the curve after decomposition by the above method is more stable than that before decomposition.

**Figure 8:**
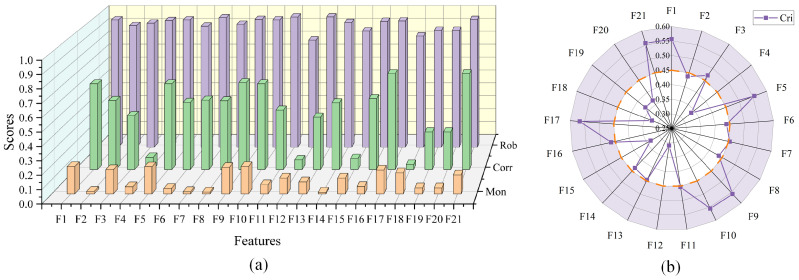
Preferred feature map.

Combining the time domain, frequency domain and improved wavelet packet energy entropy features, the original multi-domain feature set D was formed and optimized. Figure 8 is the feature evaluation index diagram. Figure 8a shows the Mon, Corr and Rob values of 21 degradation features. Then, comprehensive evaluation index Cri as shown in Figure 8b was obtained by using comprehensive evaluation index for weighting, and its average value was taken as the screening threshold A for feature screening (orange dotted circle). Ten effective features of Z≥A(A(IB2−1)=0.45) were selected to construct the preferred feature set $D^{\prime }$.

**Figure 9:**
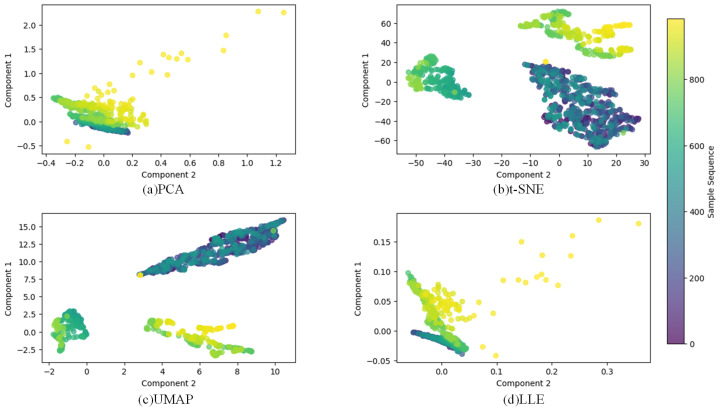
Dimensionality reduction comparison diagram.

Figure 9a–d are visualized dimensionality reduction using PCA, t-SNE, UMAP, and LLE respectively. Blue represents early or normal state, while yellow indicates near-failure or complete failure state. Based on the color trend, the position of the sample in the life cycle of the bearing can be inferred. It can be seen that compared with other dimensionality reduction techniques, UMAP dimensionality reduction can best characterize the degradation process, so using UMAP for dimensionality reduction can improve the degradation analysis ability to a certain extent.

**Figure 10:**
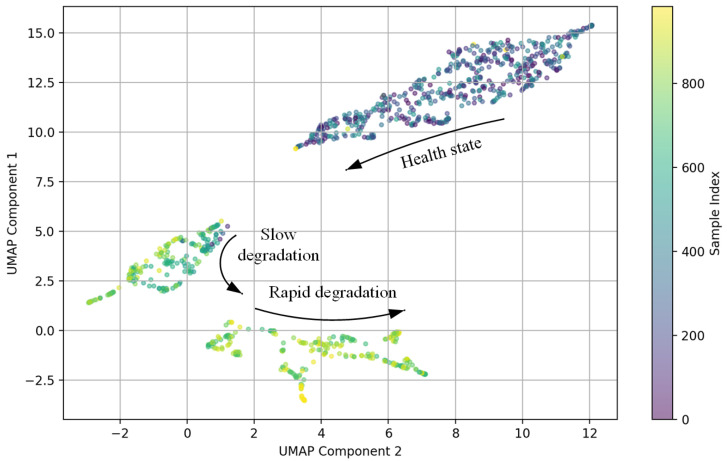
UMAP scatter plot after dimensionality reduction.

Figure 10 shows the scatterpoint magnification diagram of D’ after dimensionality reduction by UMAP. Bearings show varying degrees of degradation during their life cycle, which may be displayed as multiple clusters in the feature space.

#### 4.1.2. Establishment of Degradation Model

**Figure 11:**
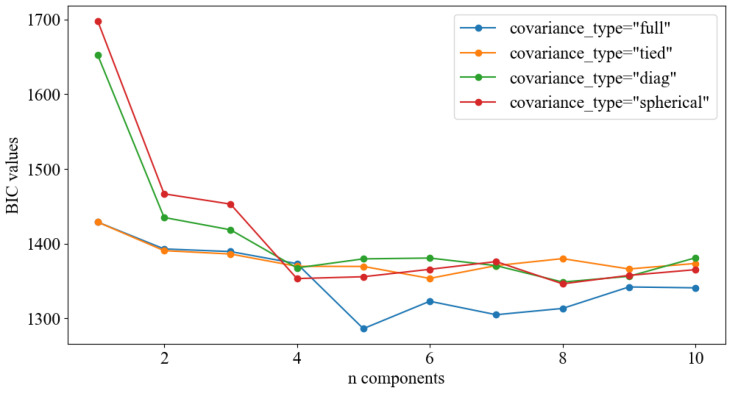
BIC of various component numbers and covariance structures.

In this paper, the first 200 trouble-free datasets were used as the training set of the GMM model. Firstly, the point with the lowest BIC score was found according to the Equation (15). As can be seen from Figure 11, when the covariance type is “full”, the overall BIC score is small. On this basis, when the mixing number is less than 5, the BIC decreases; when the mixing number is greater than 5, the BIC increases, and the optimal Gaussian mixing number is 5. It is concluded that the best covariance type is “full”, and the mixing number (n components) is 5.

**Figure 12:**
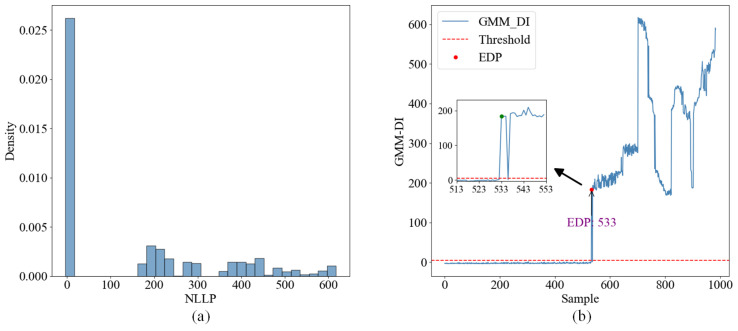
(**a**) GMM model negative log-likelihood probability density histogram; (**b**) GMM model degradation curve.

Figure 12a shows the histogram of the negative log-likelihood probability density calculated by GMM, while Figure 12b shows the corresponding negative log-likelihood probability curve. In Figure 12a, NLLP values of most data are close to zero, indicating that these are normal samples. At the same time, some data points have high NLLP values, indicating that these samples may not match the distribution of the GMM model and may be outliers. The negative log-likelihood probability curve (defined as GMM-DI) in Figure 12b is used to describe the degradation of the sample. When the abnormal discrete points are discarded and the value of three consecutive sample points is greater than the value of “threshold−1”, the first sample point is marked as EDP, that is, sample point 533 as shown in Figure 12b.

**Figure 13:**
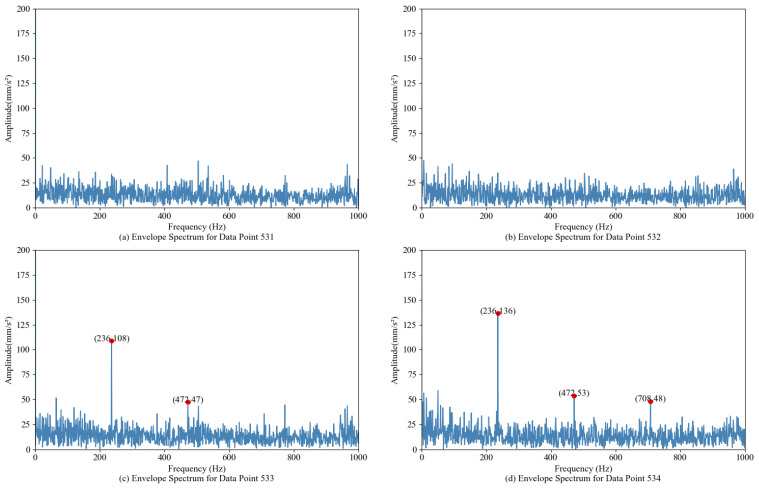
The envelope spectrum of 531–534 samples.

Envelope spectrum analysis was carried out on the original bearing vibration signal [31]. Figure 13a–d shows the envelope spectrum of the 531–534 samples. Combined with the theoretical fault characteristic frequency of bearings, it can be seen from Figure 13a,b that no component similar to the fault characteristic frequency can be found at sample points 531 and 532. On the contrary, as shown in Figure 13c,d, the 533rd sample and subsequent samples have obvious peaks at the fault frequency (236 Hz) and its frequency doubling. Early degradation occurred at sample 533 and was labeled EDP, which divided the entire bearing life into two stages—health and degradation—a conclusion consistent with the EDP found by GMM, indicating a high sensitivity of GMM to early anomalies.

However, although the above method is sensitive to early degradation, the division of subsequent degradation stages is not clear, so the introduction of distance-based method was considered for further degradation analysis. According to the FCMD method in Section 2.4.2, EDP was detected according to the definition of “threshold−1”, value. Among them, when calculating membership degree of FCM and FCMD models, combined with time domain waveform analysis as shown in Figure 5, the first 200 groups of trouble-free data and the last 10 groups of failure data were used for model training.

**Figure 14:**
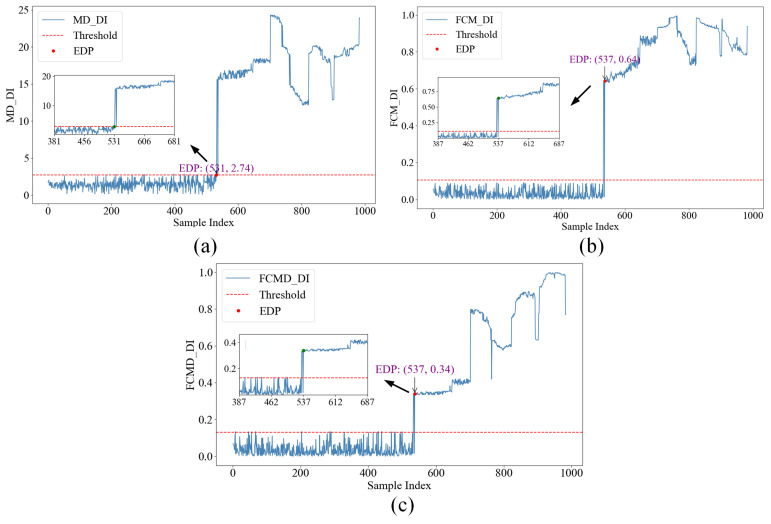
EDP detection diagram by distance method.

Figure 14a shows the degradation curve obtained by constructing the degradation index with MD. It is observed that EDP is 531 sample points; Figure 14b shows that the EDP of the membership curve that does not belong to the normal category and the threshold line is observed by FCM at 537 sample points; Figure 14c shows the degradation curve obtained by the FCMD method. It is observed that the linear intersection of the degradation index FCMD-DI and the threshold value is at 537 sample points. Combined with the envelope demodulation analysis in Figure 13, it can be seen that the three methods all have the problem of inaccurate EDP recognition. However, the degradation trend of the three methods is basically clear, especially the stage division of the degradation curve obtained by the FCMD-DI index in Figure 14c.

According to the steps of bearing degradation assessment methods in Section 3.1, combined with probability information and distance information, GMM-DI and FCMD-DI were re-input into the established FCMD model as two columns of features to obtain new fusion indexes and degradation curves. The two threshold methods of degradation detection in Section 3.2.1 can be used to determine the early degradation point and the early failure point, respectively.

**Figure 15:**
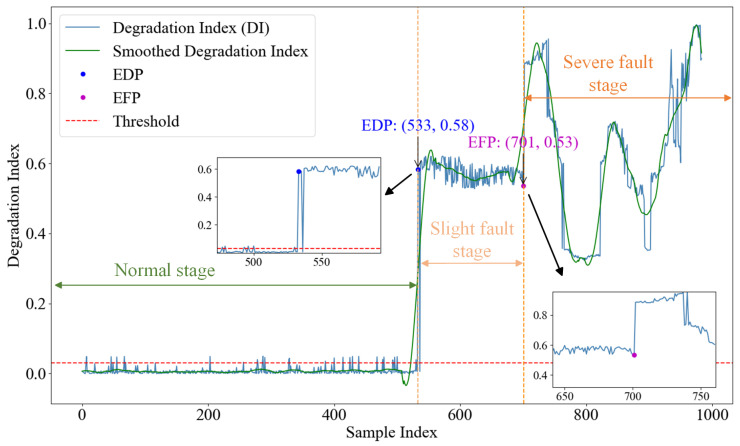
Degradation curves of the proposed method.

Figure 15 shows the degradation curve of the proposed method and its results after being treated with the exponential weighted moving average (EWMA). First, EDP was detected by “threshold−1” defined by the 3σ principle. Before sample point 533, the degradation curve was relatively stable, so the bearing was considered to be healthy and in a trouble-free state. When the bearing ran to about 533, the degradation curve showed an upward trend. The DI value of three consecutive samples exceeded the “threshold−1” value, and the bearing entered the minor fault stage, in which the bearing continued to operate and deteriorate despite its defects. Then, the GDI was calculated, and the slope change between the two mutation points can indicate the degree of bearing degradation, and the greater the slope, the faster the bearing degradation. It can be observed that the gradient changes greatly at the sample point 701, and the degradation curve rises sharply at an instant. According to the definition of “threshold−2”, the bearing begins to fail here, and the degradation curve forms a “*W*” waveform, which gradually approaches the failure state in the later stage. At this time, appropriate intervention measures are needed to prevent the sudden failure of the bearing.

**Figure 16:**
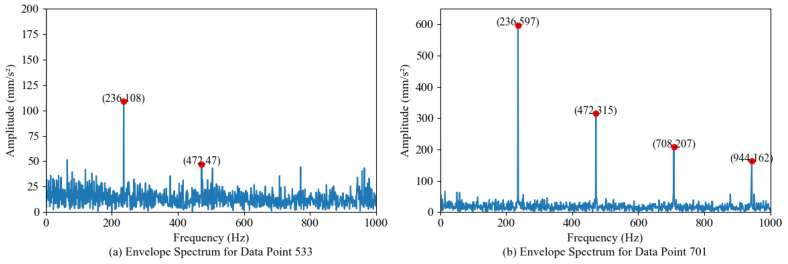
Envelope spectrum of sample points 533/701.

Similarly, we carried out envelope spectrum analysis on the sample points. Figure 16a shows the envelope spectrum of the 533rd sample point. After the vibration signal is demodulated by Hilbert envelope, the characteristic frequency and frequency doubling of the bearing outer ring fault can be observed, indicating that the bearing has degraded. In Figure 16b, the fault characteristic frequency at sample point 701 and its frequency doubling $\left(f_{0},2f_{0},\ldots \right)$ are shown. It becomes increasingly clear that the frequency amplitude is more than 500 mm/s^2^ indicating that the bearing has begun to fail. The detection results of the proposed method are highly consistent with those of envelope spectrum analysis, and the degradation point and fault point of bearings can be accurately identified, which fully verifies the effectiveness of the proposed method in practical engineering monitoring.

#### 4.1.3. Comparison of EDP Identification Results of Different Algorithms in IMS

**Figure 17:**
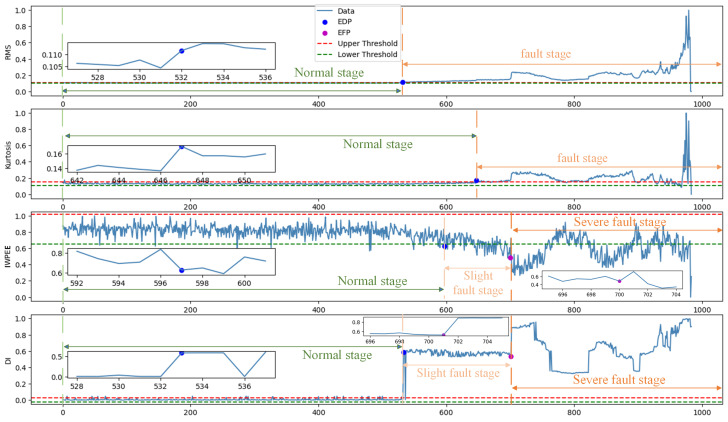
Degradation curves of different indicators.

In order to verify the superiority of the proposed method, a comparison is made with other methods, as shown in Figure 17.

Specifically, the degradation index (DI) constructed by the proposed method is compared with common DIs (RMS and kurtosis) and the DI constructed by the improved wavelet packet proposed in this paper (IWPEE). The findings are as follows:

(1) As shown in Figure 17a–c, RMS and kurtosis increase monotonically over time but are not sensitive enough to the start of degradation, with the EDP identified at 532 and 647, respectively. IWPEE also fails to accurately identify the EDP and shows significant fluctuations in the fault phase. These three DIs are not able to effectively distinguish between the normal, slight fault, and severe fault stages, nor can they accurately describe the degradation trend.
(2) According to the two threshold strategies used to analyze the proposed DI, the EDP is identified at sample 533, marking the entry into the slight fault stage, with the severe fault stage starting at sample 701. The proposed method not only accurately identifies the EDP but also better distinguishes between different degradation stages. The degradation curve exhibits an ideal monotonic trend, increasing at different rates as degradation progresses, which is similar to real-world engineering scenarios where the bearing experiences cycles of degradation, recovery, and further degradation until failure.

Therefore, the proposed method demonstrates both applicability and superiority.

In order to verify the effectiveness of the proposed method, this paper compares it with the results of some existing studies. Table 4 lists the EDP identification results of three bearings (IB1-3, IB2-1 and IB3-3) in the IMS dataset. It can be seen that the proposed method has a good effect on EDP identification, showing certain advantages. Compared with other methods, the proposed method can effectively evaluate the degradation of rolling bearings and can stably identify reasonable EDP points even under different bearing and operating conditions.

Although some of the references in Table 4 are sensitive to early degradation in specific situations, these methods perform inconsistently under different conditions and may pose a risk of instability or over-sensitivity in practical engineering applications that could affect the reliability of the system. For example, in reference [40], IB2-1 identified EDP as 530, but after in-depth analysis, it was found that this result was not accurate. Therefore, the proposed method shows higher stability and accuracy in different scenarios and is more suitable for practical engineering applications.

In addition, the literature does not provide results for other key evaluation indicators, such as monotonicity and trend, which are critical for evaluating degradation in practical engineering applications. Therefore, the comparative analysis based solely on EDP recognition results cannot fully evaluate the comprehensive performance of the proposed method, especially in engineering applications, where the monotonicity and trend of the degradation curve are key factors to ensure reliability and accuracy.

#### 4.1.4. IMS Dataset DI Performance Evaluation

**Figure 18:**
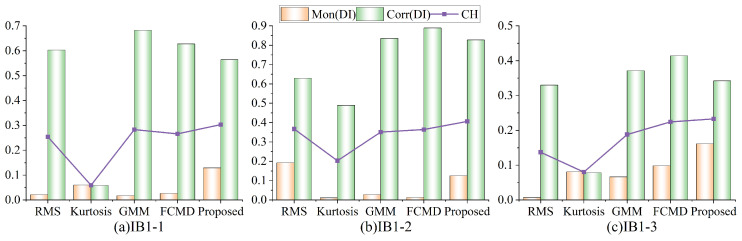
DI evaluation index for the IMS dataset.

In order to comprehensively evaluate the performance of degradation assessment methods, the monotonicity and trend of different degradation indicators are quantitatively compared. CH is a composite indicator, which is mainly used to judge the performance of DI in this study. Monotonicity is more of a concern here, so $m_{1}$ and $m_{2}$ are set to 0.6 and 0.4, respectively. Table 5 shows the calculation results of monotonicity, trend and CH of the above three bearings, and Figure 18 shows the comparison diagram of the corresponding results.

Based on the analysis of Table 5 and Figure 18, the CH scores of DI of the above three bearings are calculated by the formula in Section 3.2.2: IB1-3 is 0.303, IB2-1 is 0.406, and IB3-3 is 0.233. Compared with the traditional indexes RMS, kurtosis, probabilistic GMM and distance FCMD, DI constructed by the proposed method achieves the best results in CH score, indicating that the proposed method has good monotonicity and trend. It is worth noting that just having high monotonicity or high trend does not mean that the degradation assessment method has absolute advantages; therefore, the importance of the comprehensive index CH cannot be ignored.

In summary, the proposed method is not only sensitive to the initial degradation, it can also effectively identify EDP, clearly distinguish different degradation stages, and accurately describe the operating state and degradation trend of bearings. In the three bearing experiments, the proposed method outperforms the other four methods in CH score, indicating that its generalization performance is good, not only on the training data, but also on the new data. Therefore, the proposed method performs well in a comprehensive manner.

### 4.2. Experiment 2

**Figure 19:**
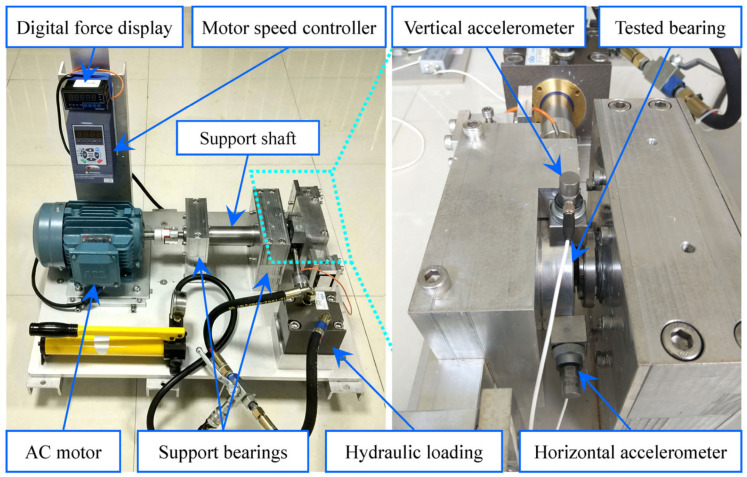
The rolling bearing test rig of XJTU-SY.

The XJTU-SY experimental bearing accelerated degradation test platform [30] is shown in Figure 19. Its sampling frequency was 25.6 kHz, sampling interval was 1min, each sampling duration was 1.28 s, and the theoretical fault frequency $f_{0}$ of the outer ring was 107.91 Hz.

Horizontal and vertical signals were acquired by multiple accelerators, and only horizontal vibration signals were considered here because radial forces were applied to the bearing under test. The test bearing model was LDK UER204. Three different working conditions were set up in the accelerated degradation experiment, and five bearings were tested under each working condition. The horizontal vibration signals of 15 bearings in the whole life cycle under three working conditions were selected for work, and the training set of each bearing experiment was set in combination with the original vibration signals, as shown in Table 6.

Due to space constraints, the process of feature extraction, selection, and dimensionality reduction was not presented here.

#### 4.2.1. Comparison of EDP Identification Results of Different Algorithms in XJTU-SY

The EDP recognition results of the XJTU-SY bearing dataset are shown in Table 7. By comparing the research results of some existing relevant studies with the method presented in this paper, it can be seen that all 15 bearings can effectively identify EDP, which is of great significance for timely detection of weak signs of degradation and prevention of major failures.

**Figure A1:**
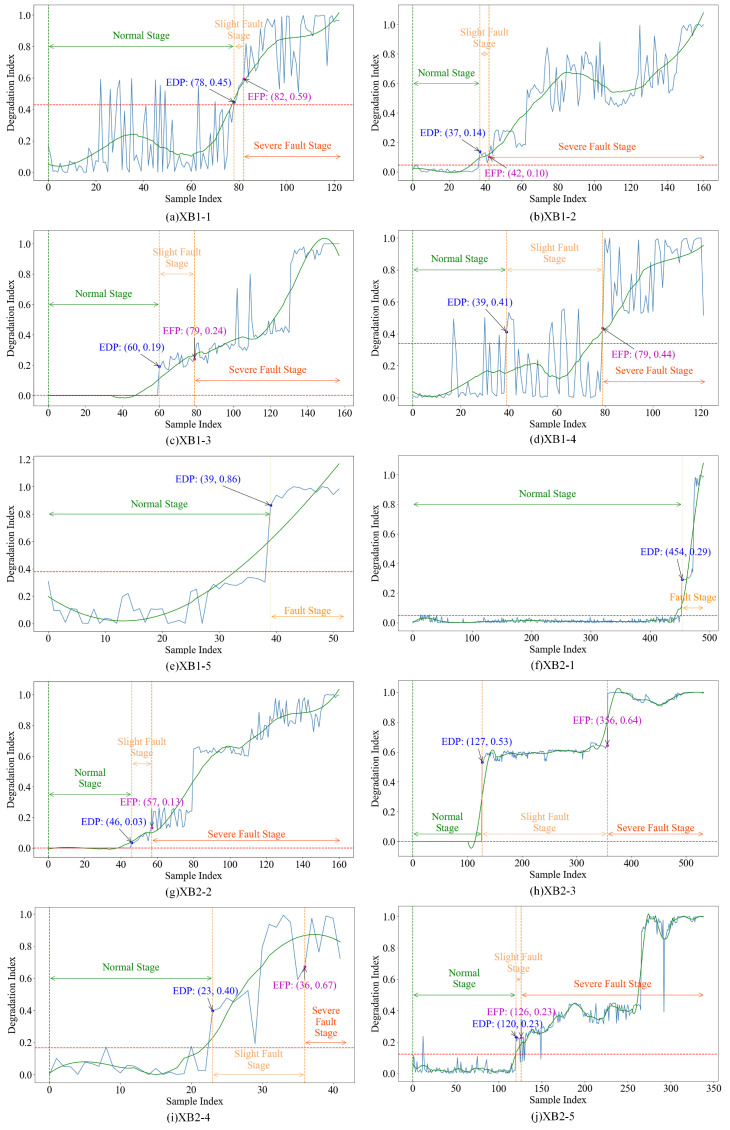

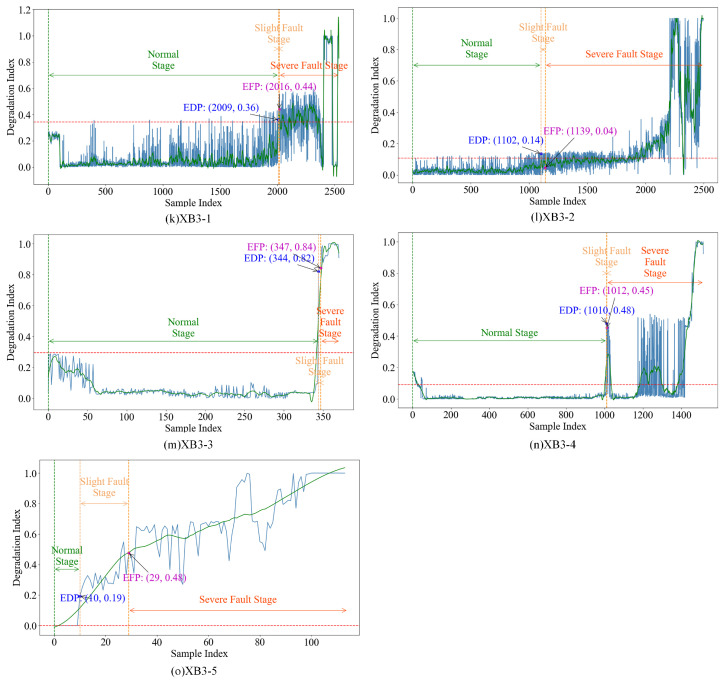
Degradation curve of XJTU-SY bearings.

The degradation curves of all 15 bearings are shown in Figure A1, and it can be seen from the figure that these curves show an obvious upward trend. This shows that the proposed method can effectively capture the degradation characteristics of bearings, quantitatively represent the severity of degradation, and fully reflect the degradation process.

#### 4.2.2. XJTU-SY Dataset Experimental Analysis

**Figure 20:**
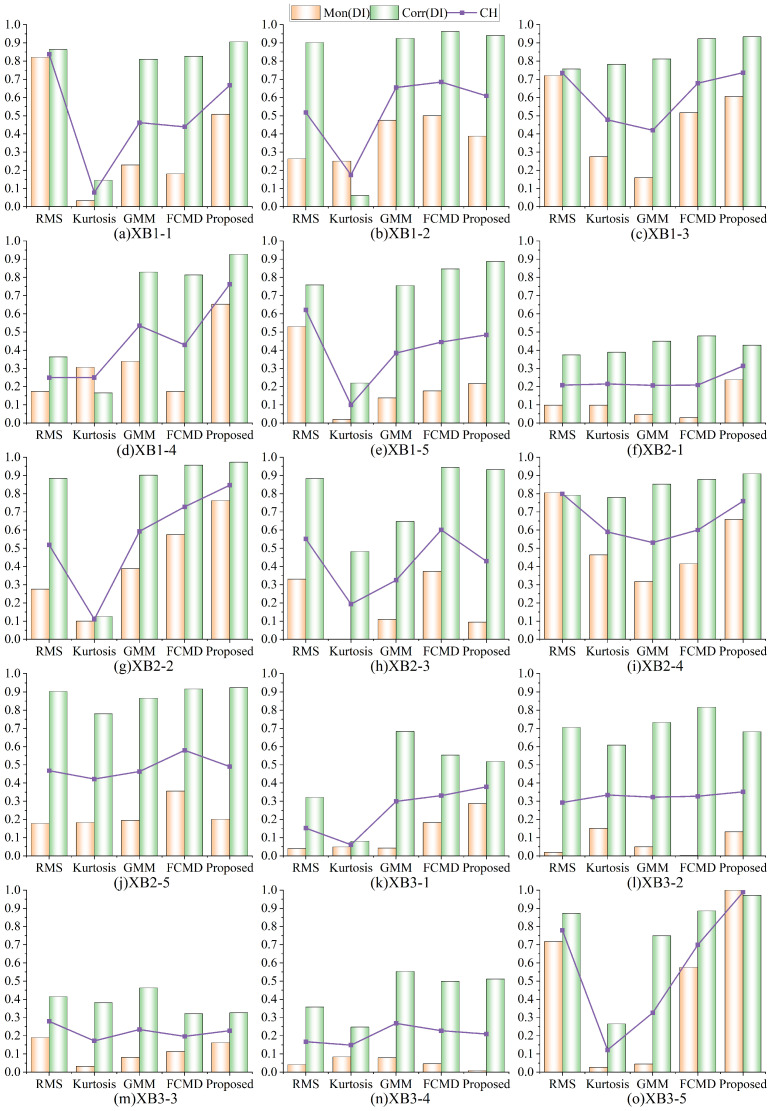
DI evaluation index for the XJTU-SY dataset.

Similarly, in order to verify the superiority of the proposed method, this paper compared it with the other four methods, calculated the evaluation index, and still set $m_{1}$ and $m_{2}$ as 0.6 and 0.4, respectively. Figure 20 shows the monotonicity, trend of DI and the score of CH comprehensive evaluation index constructed by different methods.

It can be seen that the proposed method ranks first in the CH score index for many times, that is, the best results are obtained in Figure 20c,d,f,g,k,l,o, which is superior to other methods. Compared with a single index, the comprehensive evaluation score is higher and the monotonicity trend is better. Therefore, the proposed method performs stably and maintains a leading position in multiple experiments, with wider applicability and effectiveness. In general, the proposed method performs well in all aspects. It is sensitive to initial degradation and can accurately detect EDP, and also, the DI shows good monotonicity and trend, performs better in the whole degradation trend, and has better performance compared with other methods.

## 5. Conclusions

In order to solve the problem of randomness in the spatial distribution of bearing degradation characteristics under heavy load conditions, an innovative evaluation method integrating multi-domain features and deep fusion degradation model was proposed and applied in this paper. This method can fully reflect the degree of bearing degradation and effectively improve the ability to capture multi-dimensional information in the process of degradation. The precision labeling of early degradation points is realized, and the degradation trend can be dynamically tracked, which significantly improves the accuracy of degradation state assessment and effectively solves the problem of insufficient sensitivity and accuracy in degradation identification by traditional methods. The validation on IMS and XJTU-SY datasets shows that the proposed method has excellent performance in early degradation identification and trend tracking, is reasonable, effective and comprehensive, and can accurately quantify the bearing degradation state. At the same time, it breaks through the problem of insufficient adaptability of traditional methods under complex working conditions, which is of great significance for timely detection of degradation signs and prevention of major failures, and provides a scientific basis for predictive maintenance of equipment.

Compared with the previous methods, although the training time and the complexity of feature extraction are increased, the performance of the current method is significantly improved. Future work can focus on further optimizing the evaluation method to improve the adaptability and efficiency of the algorithm under complex working conditions and exploring the application of online monitoring to enhance the early warning ability in industrial practice. The application and scenario data should be collected continuously, and the practicability of the method needs to be verified on the actual large-scale engineering data set.

## Figures and Tables

**Table 1:** Time domain characteristic formula.

Features	Formula	Features	Formula
$x_{rms}$	$F_{1}=\sqrt{\frac{1}{N}\sum_{i=1}^{N}x_{i}^{2}}$	$|\bar{x}|$	$F_{9}=\frac{1}{N}\sum_{i=1}^{N}\left|x_{i}\right|$
$x_{p}$	$F_{2}=max\left|x_{i}\right|$	$x_{r}$	$F_{10}={\left(\frac{\sum_{i=1}^{N}\sqrt{|x_{i}}|}{N}\right)}^{2}$
$x_{\delta }$	$F_{3}=\frac{\sum_{i=1}^{N}{\left(x_{i}-\bar{x}\right)}^{2}}{N}$	$s_{f}$	$F_{11}=\frac{F_{1}}{F_{9}}$
$\bar{x}$	$F_{4}=\frac{1}{N}\sum_{i=1}^{N}x_{i}$	$c_{f}$	$F_{12}=\frac{F_{2}}{F_{1}}$
$x_{{rms}^{2}}$	$F_{5}=\frac{1}{N}\sum_{i=1}^{N}x_{i}^{2}$	$I_{f}$	$F_{13}=\frac{F_{2}}{F_{9}}$
$x_{max}$	$F_{6}=max\left\{x_{i}\right\}$	$K_{r}$	$F_{14}=\frac{\frac{1}{N}\sum_{i=1}^{N}x_{i}^{4}}{F_{1}^{4}}$
$x_{min}$	$F_{7}=min\left\{x_{i}\right\}$	$x_{f}$	$F_{15}=\frac{F_{2}}{F_{10}}$
$x_{p-p}$	$F_{8}=F_{6}-F_{7}$	$c_{w}$	$F_{16}=\frac{\frac{1}{N}\sum_{i=1}^{N}x_{i}^{3}}{F_{1}^{3}}$

**Table 2:** Frequency domain characteristic formula.

Features	Formula	Features	Formula
$F_{m}$	$F_{17}=\frac{\sum_{k=1}^{N}s\left(k\right)}{N}$	$F_{rms}$	$F_{19}=\sqrt{\frac{\sum_{N}^{k=1}f_{k}^{2}s\left(k\right)}{\sum_{N}^{k=1}s\left(k\right)}}$
$F_{c}$	$F_{18}=\frac{\sum_{N}^{k=1}f_{k}s\left(k\right)}{\sum_{N}^{k=1}s\left(k\right)}$	$F_{sd}$	$F_{20}=\sqrt{\frac{\sum_{N}^{k=1}{\left(f_{k}-F_{18}\right)}^{2}s\left(k\right)}{\sum_{N}^{k=1}s\left(k\right)}}$

**Table 3:** Description of IMS dataset.

Bearing Number	File Number	Channel	Fault Location	Training Set	Test Set
IB1-3	2156	5	Inner ring fault	1000	2156
IB2-1	984	1	Outer ring fault	200	984
IB3-3	6324	3	Outer ring fault	2000	6324

**Table 4:** Comparison table of EDP for IMS.

Bearings	RMS	Kurtosis	FCMD	GMM	Method in [38]	Method in [39]	Method in [40]	Method in [41]	Method in [30]	Proposed
IB1-3	2117	1830	1712	1715	2120	1796	1999	1786	1859	1715
IB2-1	532	647	531	533	610	532	530	533	533	533
IB3-3	5937	6161	5980	5980	2435	5973	/	/	6001	5980

**Table 5:** Evaluation index of IMS bearings.

Bearings	Method	*Mon* (*DI*)	*Corr* (*DI*)	*CH*
IB1-3	RMS	0.021	0.603	0.254
	Kurtosis	0.060	0.058	0.059
	GMM	0.017	**0.682**	0.283
	FCMD	0.026	0.627	0.266
	Proposed	**0.129**	0.564	**0.303**
IB2-1	RMS	**0.192**	0.629	0.367
	Kurtosis	0.011	0.489	0.203
	GMM	0.027	0.835	0.351
	FCMD	0.013	**0.889**	0.364
	Proposed	0.125	0.827	**0.406**
IB1-3	RMS	0.007	0.330	0.137
	Kurtosis	0.081	0.078	0.080
	GMM	0.066	0.371	0.188
	FCMD	0.098	**0.414**	0.224
	Proposed	**0.161**	0.342	**0.233**

**Bold** values represent the best performance among methods for each evaluation index.

**Table 6:** Description of XJTU-SY dataset.

Condition	Bearing Number	File Number	Fault Location	Training Set	Test Set
35 Hz/12 kN	XB1-1	123	Outer ring fault	30	123
	XB1-2	161	Outer ring fault	30	161
	XB1-3	158	Outer ring fault	30	158
	XB1-4	122	Cage fault	20	122
	XB1-5	52	The inner and outer rings are faulty	30	52
37.5 Hz/11 kN	XB2-1	491	Inner ring fault	300	491
	XB2-2	161	Outer ring fault	30	161
	XB2-3	533	Cage fault	100	533
	XB2-4	42	Outer ring fault	20	42
	XB2-5	339	Outer ring fault	100	339
40 Hz/10 kN	XB3-1	2538	Outer ring fault	2000	2538
	XB3-2	2496	Inner ring, outer ring, rolling element, cage failure	1000	2496
	XB3-3	371	Inner ring fault	300	371
	XB3-4	1515	Inner ring fault	1000	1515
	XB3-5	114	Outer ring fault	10	114

**Table 7:** Comparison table of EDP for XJTU-SY.

Bearings	RMS	Kurtosis	FCMD	GMM	Method in [42]	Method in [43]	Method in [44]	Method in [45]	Method in [30]	Proposed
XB1-1	72	75	78	78	66	79	75	78	70	78
XB1-2	35	43	42	42	41	32	53	36	37	37
XB1-3	58	59	59	60	65	59	103	58	59	60
XB1-4	83	85	47	39	10	81	117	29	80	39
XB1-5	31	48	39	39	31	36	31	/	36	39
XB2-1	452	454	454	454	390	453	451	452	455	454
XB2-2	46	47	46	46	47	47	85	46	47	46
XB2-3	127	127	126	127	260	127	323	302	315	127
XB2-4	30	31	23	23	27	31	27	121	31	23
XB2-5	121	138	120	120	130	122	161	/	122	120
XB3-1	2339	1588	2022	2046	2059	2377	2333	2346	2378	2009
XB3-2	1231	895	1229	1229	147	/	2211	340	2069	1102
XB3-3	341	339	344	344	294	341	341	1417	344	344
XB3-4	1417	1417	1010	1418	1217	1418	1435	10	1418	1010
XB3-5	10	13	10	10	10	7	33	/	5	10

## Data Availability

The original contributions presented in this study are included in the article. Further inquiries can be directed to the corresponding author.

## Abbreviations

The following abbreviations are used in this manuscript:

DI	degradation index
EDP	early degradation point
RMS	root mean square
GMM	Gaussian mixture distribution model
NLLP	negative log-likelihood probability
MD	Mahalanobis distance
JS	Jensen–Shannon
FCM	fuzzy C-mean
FCMD	fuzzy c-means algorithm based on Mahalanobis distance
WPD	wavelet packet decomposition
WPEE	wavelet packet energy entropy
EFP	early failure point
$x_{rms}$	root mean square of signal
$x_{p}$	peak amplitude
$x_{\delta }$	variance
$\bar{x}$	mean value
$x_{{rms}^{2}}$	mean square value
$x_{max}$	maximum value
$x_{min}$	minimum value
$x_{p-p}$	peak-to-peak amplitude
$|\bar{x}|$	mean amplitude
$x_{r}$	RMS amplitude
$s_{f}$	shape factor
$c_{f}$	Peak factor
$I_{f}$	Impulsion factor
$K_{r}$	kurtosis
$x_{f}$	clearance factor
$c_{w}$	skewness
$F_{m}$	frequency mean
$F_{c}$	centroid frequency
$F_{rms}$	RMS frequency
$F_{sd}$	frequency standard deviation
